# Global epidemiology and resistance-related mutations of ceftazidime-avibactam-resistant *Klebsiella pneumoniae* strains

**DOI:** 10.3389/fcimb.2025.1645042

**Published:** 2025-09-25

**Authors:** Xinya Xie, Jiali Chen, Lihua Qi, Yue Yuan, Jiamin Long, Xuelian Wu, Yuan Liu, Jinpeng Guo, Changjun Wang, Xiangzhao Meng, Xiong Liu, Yong Chen, Jie Liu

**Affiliations:** ^1^ School of Public Health, China Medical University, Shenyang, China; ^2^ Chinese People’s Liberation Army (PLA) Center for Disease Control and Prevention, Beijing, China; ^3^ School of Medicine, NanKai University, Tianjin, China; ^4^ Department of Laboratory, The Seventh Medical Center of Chinese People’s Liberation Army (PLA) General Hospital, Beijing, China; ^5^ Department of Disease Control and Prevention, The Fifth Medical Center of Chinese People’s Liberation Army (PLA) General Hospital, Beijing, China; ^6^ Department of Laboratory, Beijing Aerospace General Hospital, Beijing, China; ^7^ The Second School of Clinical Medicine, Southern Medical University, Guangzhou, China

**Keywords:** *Klebsiella pneumoniae*, ceftazidime-avibactam resistance, resistance-related mutations, genome analysis, outer membrane porins

## Abstract

**Objective:**

Carbapenem-resistant *Klebsiella pneumoniae* (CRKP) infection has become a global public health problem in recent years. However, ceftazidime-avibactam-resistant (CAZ/AVI-R) *Klebsiella pneumoniae* (*K. pneumoniae*) has emerged during treatment for CRKP infection. Therefore, understanding the molecular epidemiological characteristics and resistance-related mutations of global CAZ/AVI-R *K. pneumoniae* strains is crucial for guiding the rational use of CAZ/AVI and for implementing the control measures to prevent the spread of CAZ/AVI-R *K. pneumoniae*.

**Methods:**

Non-repetitive *K. pneumoniae* strains isolated from clinical and sewage samples from three hospitals were subjected to antimicrobial susceptibility testing (AST) and whole-genome sequencing (WGS). According to the E-test results, 37 and 11 CAZ/AVI-R *K. pneumoniae* strains were included from clinical and sewage samples, respectively. After applying the inclusion and exclusion criteria and carrying quality control, 253 CAZ/AVI-R *K. pneumoniae* strains with genome sequences were retrieved from public databases. Sequence types (STs) and serotypes were identified using Kleborate. Antimicrobial resistance genes (ARGs), virulence factors, and plasmids were annotated using Abricate. Insertion sequences (ISs), prophages, and macromolecular secretion systems were predicted using ISEScan, Dbscan, and Macsyfinder, respectively. Prokka, Roary, and IQtree2 were used for annotation, core gene alignment, and phylogenetic analysis, respectively. Mutations in outer membrane porins (*OmpK35* and *OmpK36*) and efflux pumps (*AcrA* and *AcrB*) were analyzed and visualized using Miniprot and BioAider.

**Results:**

A total of 301 CAZ/AVI-R *K. pneumoniae* strains were collected, comprising 37 human clinical strains, 11 sewage-derived strains, and 253 strains from public databases. Through comprehensive annotation, we identified 43 STs, 37 capsular (K) serotypes, 5 O antigen serotypes, 27 plasmid types and 22 ISs, 135 virulence genes, and 10 macromolecular secretion systems were annotated. Prophages carrying ARGs were annotated in 106 strains. The phylogenetic tree was roughly divided into 10 clades based on porins mutations, ARGs, virulence scores, and plasmid types. In total, 128 distinct ARGs were identified among the 301 strains. The ARGs associated with CAZ/AVI resistance in *K. pneumoniae* strains mainly included the class B metallo-beta-lactamases (MBLs) genes, *bla*_KPC-2_, and *bla*_KPC-3_ variants. Analysis of porin mutations revealed that in *OmpK35*, the most common substitution among all strains from three collection sources was at position 28 (*aaK). *OmpK36* exhibited the highest number of mutations among strains from three sources, with frequent changes at position 136 (T136G), 137 (-gacacc), and 349 (H349R). Some porin mutations were identified exclusively in strains isolated from hospital clinical samples by our research team. *OmpK35* had a substitution at position 132 (E132K). *OmpK36* had substitutions at positions 2 (K2S), 3 (V3L), and 146 (R146H), respectively. *AcrA* and *AcrB* had substitutions at positions 188 (T188A) and 716 (R716L), respectively. Among the 301 strains, the majority had multiple drug resistance-related mutations, which were extensively distributed across 10 different clades.

**Conclusion:**

The combination of multiple drug resistance-related mutations leads to resistance to CAZ/AVI. The most common resistance-related mutations in strains from both public databases and those collected by our team are the coexistence of outer membrane porins and efflux pump mutations, and carriage of MBLs genes.

## Introduction

*Klebsiella pneumoniae* (*K. pneumoniae*), a common Gram-negative opportunistic pathogen, can cause infections across various body parts (respiratory tract, urinary tract, and bloodstream), leading to a range of diseases such as pneumonia, urinary tract infection, bacteremia (Ballén et al., 2021; Jin et al., 2022; Holmes et al., 2023; Song et al., 2024). According to the data from the China Antimicrobial Resistance Surveillance System (CARSS) in 2023, the detection rate of *K. pneumoniae* among clinical Gram-negative bacteria reached 22.8%, second only to Escherichia coli at 28.3%. The average detection rate of carbapenem-resistant *K. pneumoniae* (CRKP) in China was 10.8%, with rates ranging from 0.6% to 26.2% in different provinces (China Antimicrobial Resistance Surveillance System, 2023). The prevalence of CRKP has increased in China. The treatment of last-line agents, such as polymyxins and tigecycline, may be constrained due to resistance, unfavorable pharmacokinetics, and high toxicity rates (Paul et al., 2014; Thaden et al., 2017; Zusman et al., 2017). The effective treatment strategies for CRKP infection are limited. Consequently, the prognosis for infected patients is poor, with a high mortality rate.

A beta-lactam/beta-lactamase-inhibitor combination agent, ceftazidime-avibactam (CAZ/AVI), was approved by the U.S. Food and Drug Administration (FDA) in 2015 and was implemented in China in 2019 (Wang et al., 2022; Actavis, 2015). This has largely alleviated concerns about traditional treatment options for CRKP infection. Ceftazidime, a third-generation cephalosporin, exerts antibacterial activity by inhibiting bacterial cell wall synthesis via blocking the cross-linking of peptidoglycans (Hayes and Orr, 1983). Avibactam (AVI), on its own, has no antibacterial activity but is highly stable against *K. pneumoniae* carbapenemase (KPC) enzymes. When used in combination with broad-spectrum cephalosporins such as ceftazidime, it protects ceftazidime from hydrolysis by KPC enzymes, thereby restoring the antibacterial activity of CAZ (Testa et al., 2015). Although CAZ/AVI is a promising drug for the clinical treatment of multidrug-resistant (MDR) Gram-negative bacterial infection and has been used clinically for a short time, reports of clinical CAZ/AVI-R CRKP infection have already emerged (Chen et al., 2022; Hobson et al., 2022; Cui et al., 2023).

Therefore, it is urgent to elucidate the genome characteristics of CAZ/AVI-R CRKP. In this study, we collected the whole-genome sequence data of CAZ/AVI-R CRKP strains from previous literature and the Pathosystems Resource Integration Center (PATRIC), and our research group. By reanalyzing and summarizing the molecular characteristics of global CAZ/AVI-R CRKP strains, we aimed to delineate their epidemiological features, summarize known resistance-related mutations, and discover potential new resistance-related mutations which can provide a valuable reference for optimizing clinical treatment options.

## Materials and methods

### Settings

The Fifth Medical Center and the Seventh Medical Center of the Chinese People’s Liberation Army (PLA) General Hospital, and Beijing Aerospace General Hospital are tertiary hospitals in Beijing that integrate medical care, teaching, research, and prevention. Each hospital has thousands of beds and tens of thousands of patients every year.

### Collection of samples

Isolates were collected from clinical and hospital sewage samples. Clinical *K. pneumoniae* strains were collected from the Seventh Medical Center of the Chinese PLA General Hospital and Beijing Aerospace General Hospital. Additionally, sewage samples were collected from the Fifth Medical Center of the Chinese PLA General Hospital. The inclusion criteria for clinical isolates were as follows: 1) strains were collected from various clinical samples such as urine, blood, sputum, bronchoalveolar lavage fluid (BALF), feces, and ascites; 2) strains were identified as *K. pneumoniae* by culture; 3) the infection was monomicrobial infection by *K. pneumoniae*. Strains isolated from different infection sites of the same patient were eligible for inclusion. The exclusion criteria were: 1) repeated isolates from the same site of the same patient, only the first isolate within a two-week period was selected; 2) the infection was polymicrobial infection; 3) isolates with incomplete clinical information. Starting in July 2024, sewage samples were collected every Monday using 500 mL sterile water sampling bags. The sample collection lasted for 2 months, and a total of eight samples were obtained.

### Isolation and identification of strains

Strains were cultured on selective MacConkey agar plates containing CAZ/AVI and identified by the VITEK 2 system (BioMérieux). *Escherichia coli* ATCC25922 and *Pseudomonas aeruginosa* ATCC27853 were used as quality control strains. The E-test method was employed to assess susceptibility to CAZ/AVI, with *Klebsiella pneumoniae* ATCC700603 serving as the quality control strain. Thirty-seven and eleven CAZ/AVI-R *K. pneumoniae* strains were isolated from clinical and hospital sewage samples, respectively. The identified CAZ/AVI-R *K. pneumoniae* strains were preserved in 40% sterile glycerol solution at -80 °C for subsequent studies. Additionally, 236 CAZ/AVI-susceptible (CAZ/AVI-S) *K. pneumoniae* strains previously collected by our research group were included in this study.

### Antimicrobial susceptibility testing

According to the instructions, a Gram-negative antimicrobial susceptibility testing (AST) card (VITEK 2 AST-GN13) was used to perform the AST. *Escherichia coli* ATCC25922 and *Pseudomonas aeruginosa* ATCC27853 were used as quality control strains. The panel of 17 antibiotics tested included ampicillin/sulbactam, piperacillin/tazobactam, cefazolin, cefotetan, ceftazidime, ceftriaxone, cefepime, aztreonam, ertapenem, imipenem, meropenem, amikacin, tobramycin, ciprofloxacin, levofloxacin, nitrofurantoin, and trimethoprim/sulfamethoxazole. AST results interpretation were carried out in accordance with the Clinical and Laboratory Standards Institute (CLSI) 2023 guidelines (Clinical Laboratory Standards Institute, 2023). CRKP is defined as a *K. pneumoniae* strain that exhibits resistance to at least one of the carbapenem class antibiotics, namely imipenem, meropenem, ertapenem (doripenem is not available in China).

### DNA extraction and whole genome sequencing

Genomic DNA was extracted using a bacterial DNA extraction kit according to the manufacturer’s instructions. Sequencing was performed on the Illumina NovaSeq 6,000 platform using the NEBNext^®^ Ultra™ DNA Library Prep Kit.

### Collection of the genomic sequences from public databases

The genomic sequences of CAZ/AVI-R *K. pneumoniae* strains were retrieved from PubMed and the Pathosystems Resource Integration Center (PATRIC) database. The search strategy was: ((((Ceftazidime-Avibactam) OR (CZA)) OR (CAZ/AVI)) AND (*Klebsiella pneumoniae*)) AND (genome). A total of 157 relevant articles were retrieved in PubMed (as of March 20, 2024). From these articles, the complete genome sequences of CAZ/AVI-R *K. pneumoniae* strains were extracted. Complete genomes for CAZ/AVI-R *K. pneumoniae* strains from the PATRIC database were downloaded. Information on isolation source, host, collection date, and country was extracted for each strain.

The inclusion criteria were as follows: 1) strains from human host; 2) availability of complete basic information; 3) a confirmed CAZ/AVI resistance phenotype in *K. pneumoniae*. The exclusion criteria were: 1) strains from non-human hosts; 2) incomplete basic information; 3) inability to confirm CAZ/AVI resistance; 4) duplicate strains; and 5) whole-genome sequencing (WGS) data unavailable. After applying these criteria, 253 unique strains were retained. To further investigate the distribution characteristics of CAZ/AVI-R *K. pneumoniae* strains in the *K. pneumoniae* strains in China, we additionally collected complete genomes from 976 K*. pneumoniae* strains from China in NCBI GenBank (as of June 7, 2024).

### Bioinformatics analysis and visualization

#### Genome assembly, quality control and annotation

Quality control and assembly of genome sequences were carried out using Fastp v0.23.1 (Chen, 2023) and Shovill v1.0.0 (Wick et al., 2017), respectively. Centrifuge v1.0.3-beta was used to classify and remove contaminant sequences (Kim et al., 2016). Genome assembly quality was assessed with Quast v5.2.0 (Mikheenko et al., 2018). CheckM v1.2.2 was used to evaluate completeness and contamination of the assembled genomes (Parks et al., 2015). Kleborate v2.2.0 was used to determine the species identity (Lam et al., 2021).

#### Genetic typing, detection of virulence factors, drug resistance genes and mobile genetic element analysis

Kleborate v2.2.0 was used to identify sequence type (ST), calculate virulence score, and determine capsule locus (KL) and LPS antigen (O) types (Lam et al., 2021). Abricate v1.0.1 (https://github.com/tseemann/abricate) was used to identify ARGs, virulence factors, and plasmids (60% coverage and 90% identity) with the Resfinder (Zankari et al., 2012), the Virulence Factor Database (VFDB) (Liu et al., 2022), and PlasmidFinder (Carattoli et al., 2014) (updated August 29, 2024). The results were visualized as heat maps using TBtools (Chen et al., 2020). ISEScan v1.7.2.3 was used to identify and annotate insertion sequences (ISs) (Xie and Tang, 2017). DBSCAN-SWA (Gan et al., 2022) and MacSyFinder (Abby et al., 2024) were used to predict prophages and secretion systems, respectively.

#### Phylogenetic analysis

The bacterial genome was annotated using Prokka v1.14.6 (Seemann, 2014). The core genome was identified using Roary v3.13.0 (Page et al., 2015). The maximum likelihood (ML) tree was constructed using IQ-TREE2 v2.2.2.7 with automatic model selection and 1,000 bootstrap replicates (Minh et al., 2020). Phylogenetic trees were visualized with FigTree v1.4.4 (http://tree.bio.ed.ac.uk/software/figtree/) and iTOL (Letunic and Bork, 2024).

#### Mutation site analysis

Efflux pump-related genes (*AcrA* and *AcrB*) and outer membrane porins (*OmpK35* and *OmpK36*) have frequently been reported to be associated with CAZ/AVI-R *K. pneumoniae*. Therefore, we aligned reference protein sequences (*acrA*: WP_002892072.1; *acrB*: WP_002892069.1; *OmpK35*: CAA09665.1; *OmpK36*: ADG56549.1) (Xiong et al., 2023) to the genomes of all CAZ/AVI-R *K. pneumoniae* strains using Miniprot v0.13-r248 (Li, 2023) to identify mutations potentially affecting protein structure and function. Mutations were subsequently visualized with BioAider (Zhou et al., 2020).

## Results

### Antimicrobial susceptibility test results of 48 CAZ/AVI-R *K. pneumoniae* strains

In this study, 37 and 11 CAZ/AVI-R *K. pneumoniae* strains were isolated from clinical patients and hospital sewage samples, respectively (**Supplementary Table 1**). The AST results of these strains were shown in **Supplementary Table 2**. The resistance detection rates to four antibiotics including ampicillin/sulbactam, cefazolin, ceftazidime, and ceftriaxone were 100%. The resistance detection rates for piperacillin/tazobactam, cefotetan, cefepime, aztreonam, imipenem, trimethoprim/sulfamethoxazole, and nitrofurantoin ranged from 64% to 94%. The resistance detection rates for amikacin, tobramycin, ciprofloxacin, and levofloxacin were all below 60%, with the lowest rate being 16.67% for amikacin. Furthermore, 97.92% of these strains were identified as CRKP.

### General features of CAZ/AVI-R *K. pneumoniae* strains

We collected a total of 253 CAZ/AVI-R *K. pneumoniae* strains (**Supplementary Table 3**) that met the inclusion and exclusion criteria from relevant articles in PubMed and the PATRIC database. In addition, incorporating the 48 CAZ/AVI-R *K. pneumoniae* strains we collected, a total of 301 strains were finally used for further analysis. The genome sequences of 301 CAZ/AVI-R *K. pneumoniae* strains, 236 CAZ/AVI-S *K. pneumoniae* strains and 976 K*. pneumoniae* were employed to construct a core-genome phylogeny. As shown in **Supplementary Figure 1**, these 301 CAZ/AVI-R *K. pneumoniae* strains were dispersed across multiple clades.

**Figure 1:**
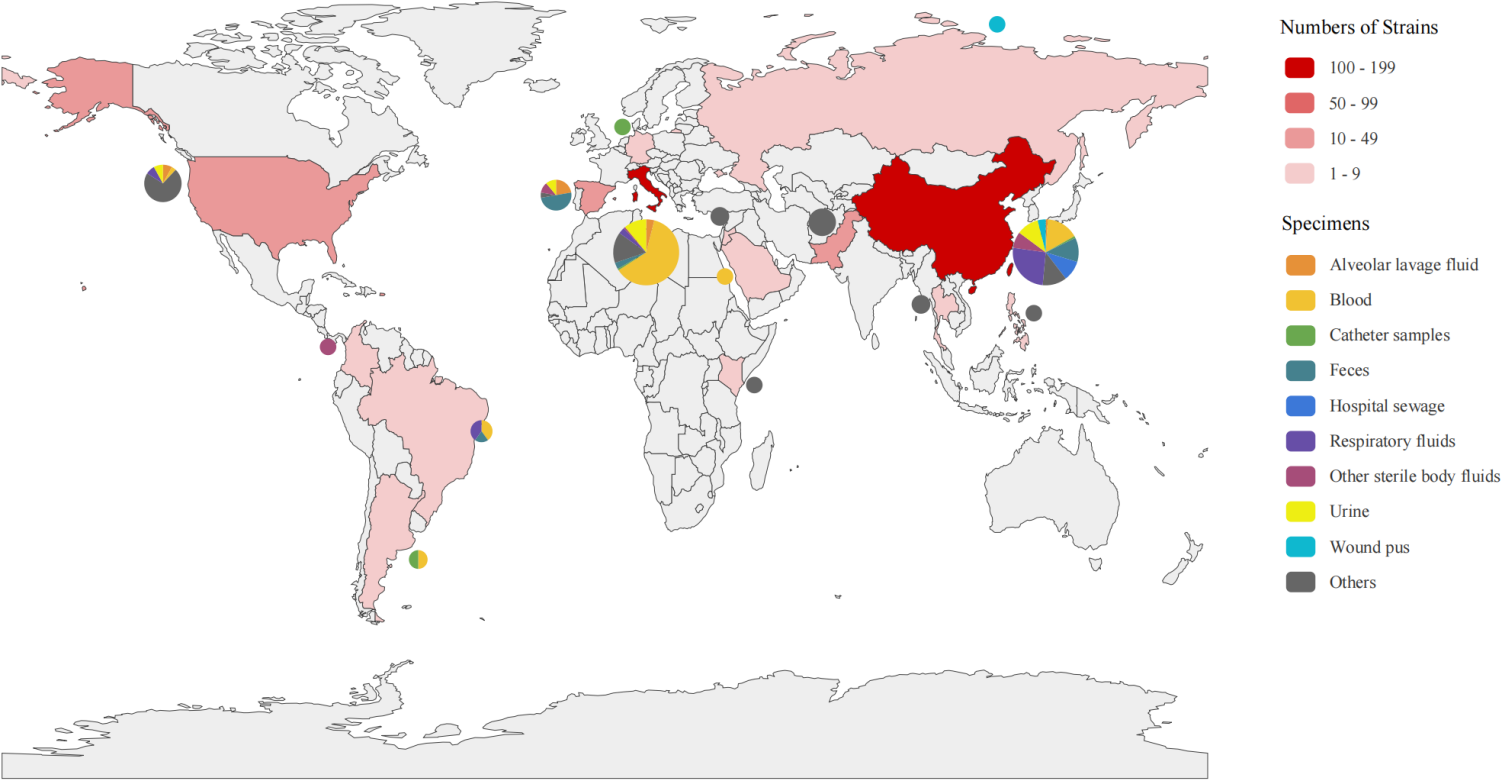
Distribution of the 301 CAZ/AVI-R *K. pneumoniae* strains. Distribution of strains collected from 15 countries worldwide. The depth of red indicates the number of strains, with darker color representing a higher number of strains. The pie charts show the isolation sources from each country.

The 301 strains were isolated from 2003 to 2024, with 75.1% of them isolated between 2018 and 2020, accounting for 75.1% of the total. They were isolated from 15 countries. Italy (41.9%), China (33.6%), the United States (8.3%), Spain (6.0%), and Pakistan (4.0%) were the main countries (**Figure 1**). There were 10 isolated sources of strains, among which blood (33.9%), respiratory secretions (11.6%), urine (11.3%) and feces (8.3%) were the most common sample types. Notably, respiratory secretions were the main source from China, particularly during 2021 (**Supplementary Figure 2**).

### Genotypic characteristics of CAZ/AVI-R *K. pneumoniae* strains

**Figure 2:**
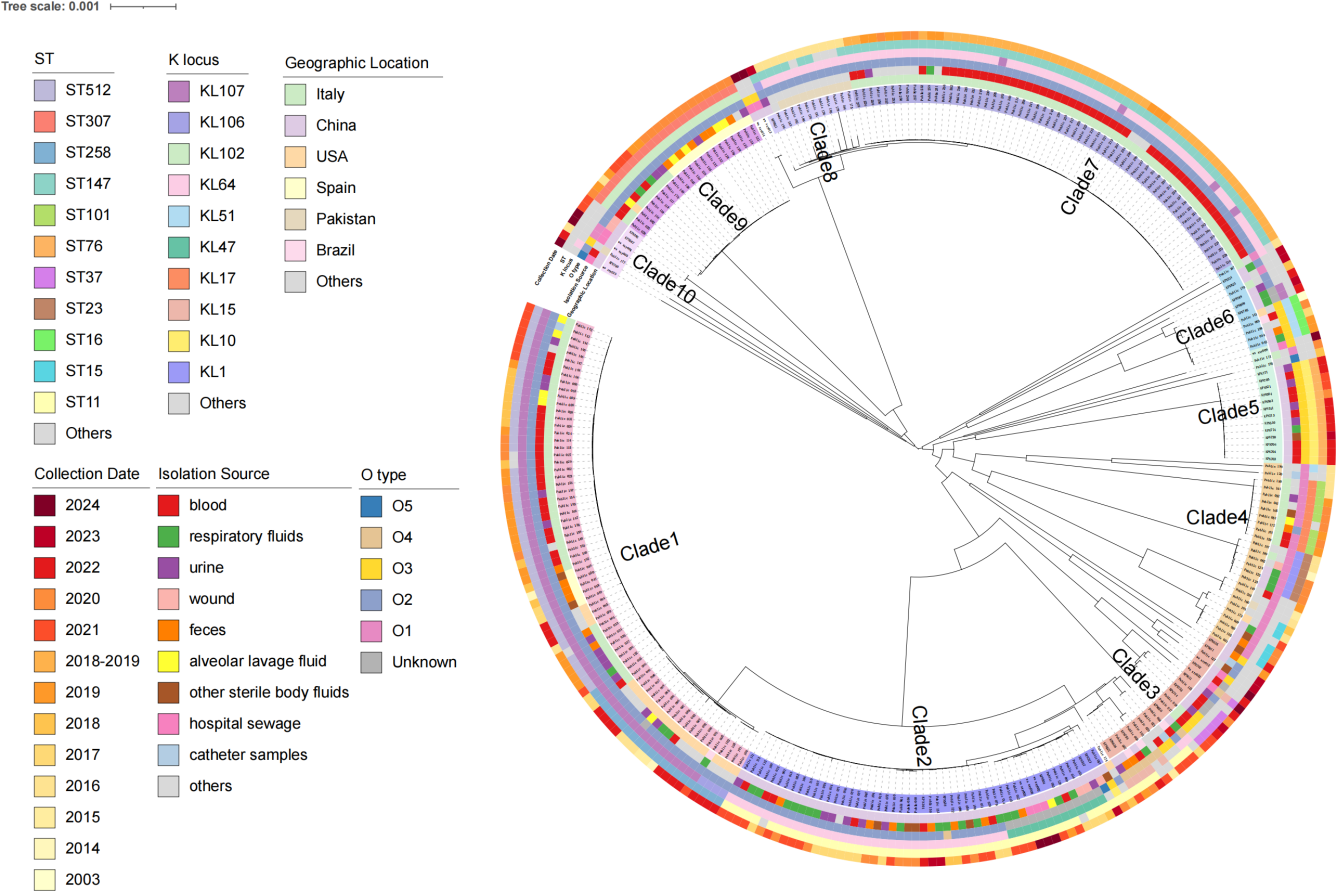
Characteristics of 301 CAZ/AVI-R *K. pneumoniae* strains based on the phylogenetic tree. They are mainly divided into 10 clades, and the strain numbers of each branch are marked with different colors. From the inner to the outer circles indicate geographic location, isolation source, O_type, K_locus, ST and collection date, respectively.

There were 43 sequence types (STs) and 37 capsular serotypes in a total of 301 strains. The main STs were ST147 (22.5%), ST11 (18.9%), ST512 (15.2%), ST307 (7.3%), and ST258 (6.9%) (**Figure 2**). The most common KL types were KL64 (35.5%), KL107 (23.9%), KL102 (7.3%), KL10 (4.6%), and KL47 (4.3%). The O antigen serotypes were predominantly O1, O2, O3, O4, and O5, with O2 being the most prevalent at 69.7% (210/301).

### Mobile genetic elements in CAZ/AVI-R *K. pneumoniae* genome

In this study, a total of 27 plasmid types were annotated, consisting of 10 colicinogenic plasmids, 14 incompatibility group plasmids, and other plasmids (repB, repB (R1701) and pKP1433) (**Supplementary Table 4**). Among all strains, 35 CAZ/AVI-R *K. pneumoniae* isolates carried no plasmids, while the remaining 266 strains harbored variable number of plasmids (ranging from 1 to 9 per strain). Twenty-two distinct insertion sequences (ISs) including IS1, IS3, IS4, IS5, IS6, IS21, IS30, IS66, IS91, IS110, IS200/IS605, IS256, IS481, IS630, IS982, IS1182, IS1380, ISAS1, ISKRA4, ISL3, ISNCY, and several new ISs were detected (**Supplementary Table 5**). The most prevalent ISs were IS3, IS5, IS21, and ISNCY. New ISs were present in 46 CAZ/AVI-R *K. pneumoniae* isolates. For the 48 CAZ/AVI-R strains collected by our team, the predicted number of ISs ranged from 5 to 12 (**Supplementary Figure 3**). Among the 301 strains, 1,692 prophages were predicted in 223 strains (**Supplementary Table 6**). Among these, 106 strains carrying prophages were annotated with 38 types of ARGs. These ARGs mainly conferred resistance to aminoglycoside, beta-lactam, chloramphenicol, trimethoprim, fosfomycin, macrolides, fluoroquinolones, sulfamethoxazole, and tetracyclines (**Supplementary Table 7**).

### Characterization of virulence genes and macromolecular secretion systems

**Figure 3:**
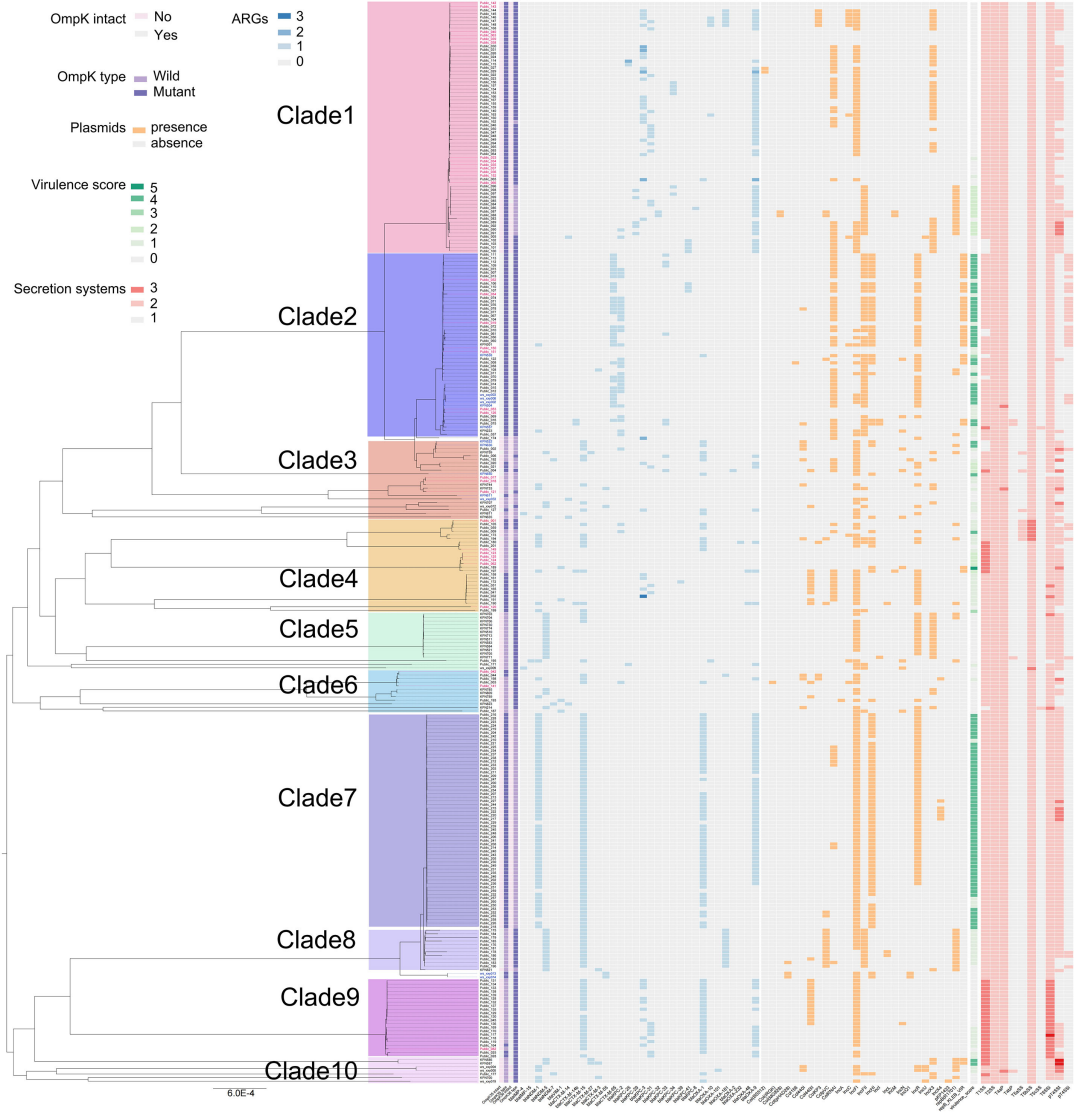
Distribution characteristics of membrane pore proteins, drug resistance genes, plasmids, virulence scores, and secretion systems of 301 CAZ/AVI-R *K. pneumoniae* strains based on the phylogenetic tree. Strain numbers in different colors indicate that they are in different clades.

A total of 135 different types of virulence genes were annotated, with each strain carrying between 49 and 132. These virulence genes were associated with regulation, antimicrobial activity/competitive advantage, immune modulation, adherence, invasion, biofilm, exotoxin, effector delivery system, and nutritional/metabolic factor (**Supplementary Figure 4A**, **Supplementary Table 8**). All strains contained a total of 10 macromolecular secretion systems, including type I secretion system (T1SS), type II secretion system (T2SS), type IVa pilus (T4aP), type IVb pilus (T4bP), type V secretion system (T5aSS, T5bSS, and T5cSS), type VI secretion system (T6SSi), and type IV secretion system (pT4SSt and pT4SSi) (**Figure 3**).

### Resistance-related mutations of CAZ/AVI-R *K. pneumoniae* strains

**Figure 4:**
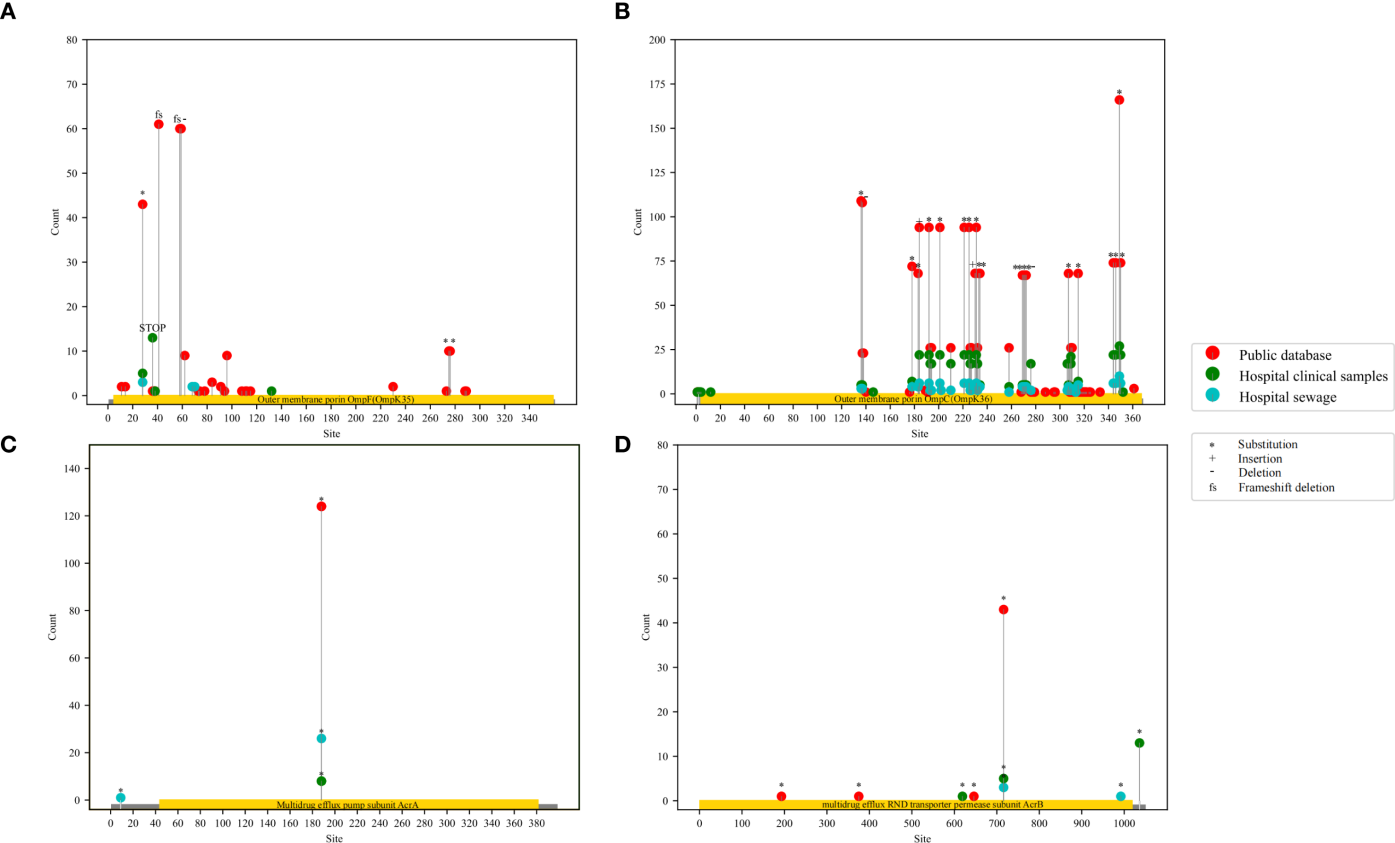
Lollipop plots of mutation sites statistics of porins and efflux pumps in 301 CAZ/AVI-R *K. pneumoniae* strains. The red dots represent CAZ/AVI-R *K. pneumoniae* strains in the public database, the green dots represent CAZ/AVI-R strains collected from hospital clinics, the blue dots represent CAZ/AVI-R strains collected from hospital sewage. “*” represents “substitution”, “+” represents insertion, “-” represents deletion, and “fs” represents frameshift mutation.

A total of 128 ARGs were annotated for 301 strains (**Supplementary Figure 4B**; **Supplementary Table 9**). We particularly focused on several ARGs associated with the mutations of CAZ/AVI resistance in *K. pneumoniae*. We found that, among the 301 strains, the class B metallo-beta-lactamases (MBLs) genes mainly included *bla*_IMP-4_, *bla*_NDM-1_, *bla*_NDM-5_, and *bla*_NDM-7_; *bla*_KPC-2_ and *bla*_KPC-3_ variants included *bla*_KPC-8_, *bla*_KPC-25_, *bla*_KPC-29_, *bla*_KPC-31_, *bla*_KPC-32_, *bla*_KPC-33_, *bla*_KPC-34_, *bla*_KPC-39_, and *bla*_KPC-41_ (**Figure 3**). The counts of mutation sites of porins and efflux pumps were shown in **Figure 4**; **Supplementary Table 10**. Most of the mutation sites of 48 CAZ/AVI-R *K. pneumoniae* strains collected in our group were the same as those of 253 CAZ/AVI-R *K. pneumoniae* strains in public databases. The most common substitution in *OmpK35* of 301 CAZ/AVI-R *K. pneumoniae* strains from the three collection sources was at position 28(*aaK). The *OmpK35* of 13 CAZ/AVI-R *K. pneumoniae* strains we collected from the clinical samples contained premature translation termination codons. The *OmpK36* had the highest number of mutations, with the most changes occurring at positions 136 (T136G: threonine was replaced by glycine), 137 (-gacacc), and 349 (H349R: histidine was replaced by arginine). Some mutations were identified exclusively in strains isolated from hospital clinical samples by our team. In the *OmpK35* porins, glutamic acid was replaced by lysine at position 132. The positions of 2, 3, and 146 of *OmpK36* porins were converted from lysine to serine (K2S), valine to leucine (V3L), and arginine to histidine (R146H), respectively. *AcrA* and *AcrB* have fewer mutations, all of which were substitutions. At position 188 of *AcrA*, threonine was replaced by alanine (T188A). At position 716 of *AcrB*, arginine was replaced by leucine (R716L).

**Figure 5:**
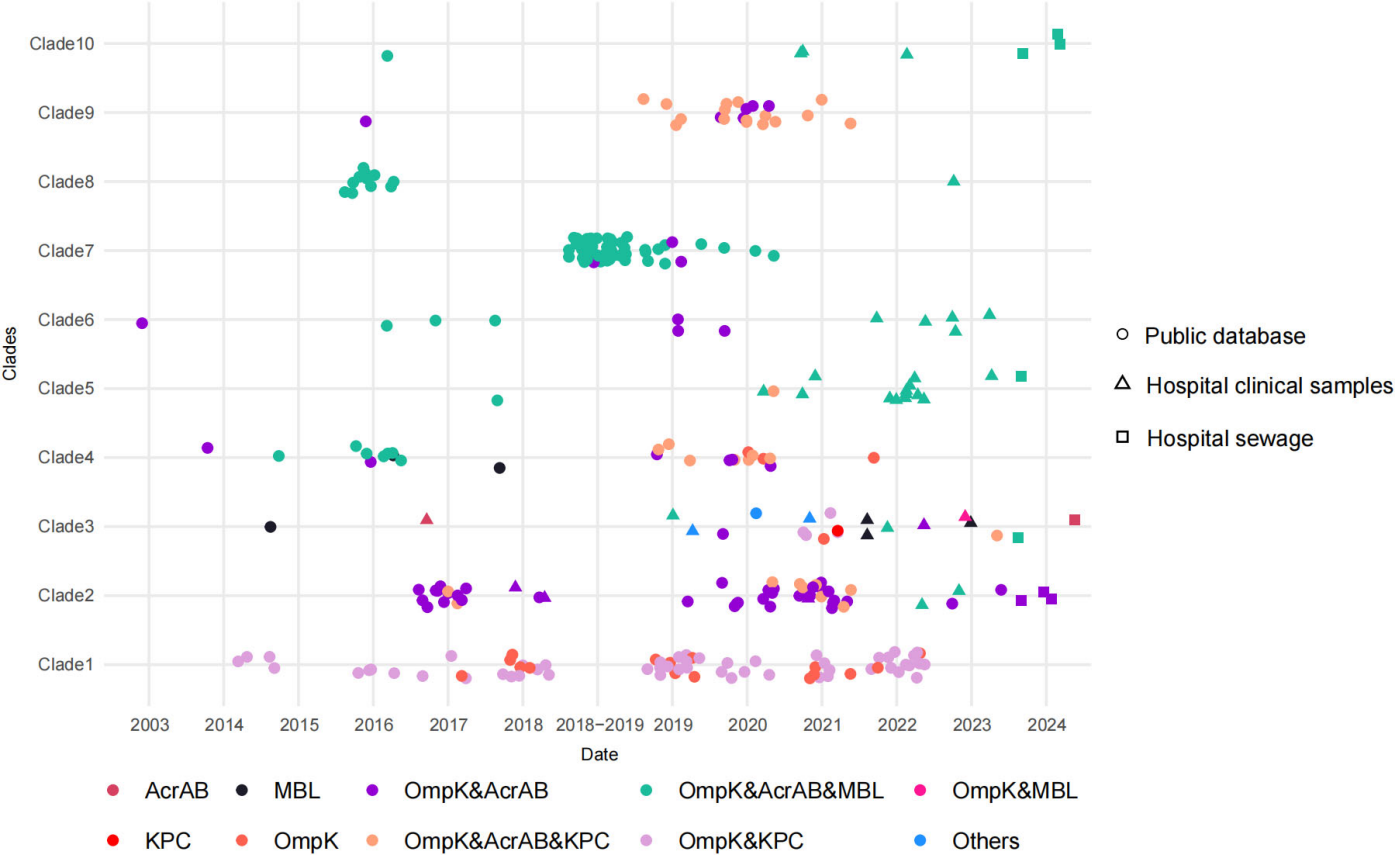
Distribution of resistance-related mutations of 301 CAZ/AVI-R *K. pneumoniae* strains. Dots, triangles and squares represent strains from public databases, hospital clinical samples, and hospital sewage, respectively. Different colors represent different resistance-related mutations.

The phylogenetic tree based on the core genome sequences of 301 CAZ/AVI-R *K. pneumoniae* strains was roughly divided into ten clades according to porins, ARGs, virulence scores, and plasmids (**Figure 3**). The strains in clade 1 were mainly characterized by mutations in porins and/or *bla*_KPC_. The strains in clade 2 were mainly characterized by mutations in porins and efflux pumps. The strains with mutations in porins, efflux pumps, and carriage of MBLs genes were distributed in clades 2–8 and clade 10. The strains in clade 9 mainly had changes in porins, efflux pumps, and/or *bla*_KPC_ (**Figure 5**).

Among the 301 strains, the coexistence of porin mutations, efflux pump mutations, and carriage of MBLs genes accounted for the highest proportion (**Supplementary Table 11**). In public databases and our collection, the proportion of CAZ/AVI-R strains caused by MBLs production showed significant differences (*χ^2^* = 27.234, *P* < 0.001). In addition, we summarized the resistance-related mutations of 48 CAZ/AVI-R *K. pneumoniae* strains collected by our research group (**Supplementary Table 12**). Two strains (KPN522 and KPN636) carried four ARGs (*bla*_CTX-M-15_, *bla*_OXA-1_, *bla*_SHV-187_, and *bla*_TEM-1B_) but none of the four resistance-related mutations we closely monitored.

### Summary of the characteristics of 10 clades in 301 CAZ/AVI-R *K. pneumoniae* strains

A total of 126 strains from Italy were distributed across seven clades, primarily in Clade 1 (43/70,61.4%) and Clade 7 (60/60, 100%). Their STs included ST147 (60 strains), ST512 (38 strains), ST1519 (3 strains), and ST258 (2 strains). Forty-seven strains and fifty-six strains were typified by KL107/O2 and KL64/O2, respectively. Most strains carried ARGs, mainly including *bla*_NDM-1_ (57 strains), *bla*_CTX-M-15_ (60 strains), *bla*_OXA-9_ (72 strains), and *bla*_OXA-1_ (53 strains). All strains had mutations in *OmpK35*. One hundred and one strains isolated from China were distributed across seven clades. All 51 strains in Clade 2 were collected from China, and each of these strains exhibited mutations in both *OmpK35* and *OmpK36*. Most of them were ST11-KL64-O2 (66.7%) and ST11-KL47 (23.5%). The virulence scores of all strains were greater than 0, and 66.7% strains had a virulence score of 4. Eighty-six point three percent of all strains in Clade 2 carried plasmids, mainly including IncFII (43 strains), ColRNAI (40 strains), and IncR (38 strains). Among the 24 strains isolated from the United States, 21 strains were in Clade 1. Most of them were ST258-KL107-O2 strains (13 strains) and harbored *bla*_OXA-9_ (19 strains). The plasmids present in the 24 strains were predominantly IncFII (14 strains) and repB (R1701) (14 strains). All 24 strains had mutations in *OmpK35*. In addition, three strains were not included in any clade.

## Discussion

CRKP was one of the most critical pathogens that poses a serious threat to human health. It was listed by the World Health Organization in February 2017 for which new antibiotics are urgently needed (World Health Organization, 2017). CAZ/AVI, as a crucial therapeutic agent, plays a significant role in treating CRKP infection. In this study, we collected genome sequences of *K. pneumoniae* strains from non-repeated clinical and sewage samples from three hospitals, as well as from previous literature and public databases. The aim was to clarify the molecular epidemiological characteristics of CAZ/AVI-R *K. pneumoniae* strains and to further identify the resistance-related mutations to CAZ/AVI.

Our research demonstrates that metallo-beta-lactamases (MBLs) are the predominant resistance-related mutations among the 48 CAZ/AVI-R *K. pneumoniae* strains. Avibactam, a novel non-beta-lactam beta-lactamase inhibitor, binds to beta-lactamases through the acylation reaction. It can effectively inhibit class A (e.g., extended-spectrum beta-lactamases, ESBLs; *K. pneumoniae* carbapenemases, KPCs), class C (e.g., AmpC), and some class D (e.g., oxacillinases, OXAs) beta-lactamase enzymes in the Ambler classification, thereby protecting ceftazidime from hydrolysis by these enzymes (Shields et al., 2015; Xu et al., 2022). However, avibactam has no inhibitory effect on MBLs (e.g., New Delhi metallo-beta-lactamase, NDM, Verona integron-encoded metallo-beta-lactamase, VIM; imipenemase, IMP). Therefore, *K. pneumoniae* strains carrying MBLs exhibit natural resistance to CAZ/AVI.

In addition to MBLs, *bla*_KPC-2_ and *bla*_KPC-3_ variants are one of the main resistance-related mutations leading to CAZ/AVI resistance in *K. pneumoniae* strains. These variants primarily enhance hydrolytic activity against CAZ or reduce affinity for AVI. In our study, we identified several variants, including *bla*_KPC-8_, *bla*_KPC-25_, *bla*_KPC-29_, *bla*_KPC-31_, *bla*_KPC-32_, *bla*_KPC-33_, *bla*_KPC-34_, *bla*_KPC-39_, and *bla*_KPC-41_. Studies have reported that, regardless of whether it is in *bla*_KPC-2_ or *bla*_KPC-3_, the most common mutation is the substitution of aspartic acid (D) with tyrosine (Y) at the 179th amino acid position, which are called new KPC mutants *bla*_KPC-33_ and *bla*_KPC-31_, respectively (Xu et al., 2022; Chou et al., 2024). This is the most frequently reported clinical mutation and the most common mutation obtained under *in vitro* CAZ/AVI screening conditions. A substitution of valine to glycine at amino acid position 240 in KPC-3 (V240G substitution of KPC-3) is commonly referred to as *bla*_KPC-8_ (García et al., 2020). The *bla*_KPC-25_ (167_168dupLE) referred as a duplication of leucine and glutamic acid at positions 167 and 168 of KPC-2 (Shen et al., 2024). The *bla*_KPC-39_ (A172T) referred as a mutation of alanine to threonine at position 172 in KPC-3 (Chou et al., 2024). The *bla*_KPC-41_ referred as an insertion of three amino acids (proline-asparagine-lysine, P-N-K) between position 269 and 270 of *bla*_KPC-3_ (Mueller et al., 2019). Overall, these mutations often occur in the Ω loop of class A beta-lactamases that is a conserved motif comprising amino acid residues from Arg164 to Asp179 of KPC (Levitt et al., 2012). These variants can reduce the susceptibility of strains to CAZ/AVI, and their emergence and spread pose a threat to public health. Therefore, continuous surveillance and research on these KPC variants in *K. pneumoniae* strains are essential.

Studies have shown that *Enterobacteriaceae* harboring pAmpC, *bla*_OXA-1_, or with (hyper) production of inhibitor-resistant *bla*_TEM_-variants, the occurrence of which is mostly reported for human clinical isolates, compromise the activity of beta-lactam/beta-lactamase inhibitor combinations (e.g., ceftolozane-tazobactam and/or CAZ/AVI) (Schechter et al., 2018; Livermore et al., 2019; Savin et al., 2021). Therefore, we will further investigate the mutations of *bla*_OXA-1_ and *bla*_TEM_-variants in CAZ/AVI-R *K. pneumoniae* strains.

Alterations in outer membrane porins and efflux pumps constitute resistance-related mutations to CAZ/AVI. In CAZ/AVI-R bacteria, porin deletions, mutations, and reduced expression are commonly observed. These alterations enhance resistance by interacting with other resistance-related mutations, such as mutations those in KPC enzymes. *OmpK35* and *OmpK36* are the most reported porins associated with CAZ/AVI-R *K. pneumoniae*. In this study, we found that *OmpK35* and *OmpK36* in CAZ/AVI-R *K. pneumoniae* exhibited varying degrees of mutation or deletion. Previous studies have demonstrated that mutations within the functional domains of proteins can affect bacterial resistance. In our study, the substitutions in *OmpK35* (E132K) and *OmpK36* (K2S, V3L, and R146H) are novel mutations discovered in our collected strains. We found no relevant reports in previous literature after a thorough review. As these novel mutations are located within the functional domains of the proteins, we speculate that they may influence the resistance of *K. pneumoniae* strains to CAZ/AVI. If such mutations are detected in future clinical isolates, close monitoring and appropriate medication use would be warranted.

The resistance-nodulation-cell division (RND) efflux pump AcrAB-TolC is composed of a periplasmic fusion protein *AcrA*, a plasma membrane transporter *AcrB*, and an outer membrane channel protein TolC. It plays an important role in the intrinsic and acquired resistance of Gram-negative bacteria (Jang and Jang, 2023). Compared with the reference sequence of *AcrB*, the most common change in *AcrB* in our study was the substitution of arginine (R) with leucine (L). A study reported that the azithromycin-resistant *Salmonella typhi* strain in Nepal did not carry a resistance gene but instead had a non-synonymous mutation in the *acrB* gene (STY0519), which changed arginine (R) at codon 717 to leucine (L) (Duy et al., 2020). The azithromycin resistance of the strain was mediated by the chromosomal mutation R717L in the *acrB* gene. This finding highlights the potential role of the *acrB* gene in mediating resistance. Furthermore, increased efflux pump activity and high expression of certain genes can contribute to bacterial resistance to CAZ/AVI. Nelson et al. found that mutations in *ramR*, a regulator of the *AcrAB* efflux pump, can lead to overexpression of the AcrAB-TolC efflux system and, together with porins alterations, promote resistance to CAZ/AVI (Nelson et al., 2017).

Interestingly, the CAZ/AVI-R *K. pneumoniae* strain (Public_141) we collected from a previous study, was isolated from a patient in New York City in 2003 (Niu et al., 2020). The original study reported that the strain carrying *bla*_KPC-14_ was sensitive to carbapenems and resistant to CAZ/AVI before the approval of CAZ/AVI in 2015. However, by reanalyzing the genome using ResFinder and BLASTn, we found that Public_141 strain had no *bla*_KPC-14_ compared to the reference sequence. This finding indicates that some KPC variants can be free from the selection pressure of CAZ/AVI, highlighting the remarkable adaptability of *K. pneumoniae*. Among the strains we collected, three strains without harboring MBLs or KPC-2 and KPC-3 variants exhibited CAZ/AVI resistance prior to the launch of CAZ/AVI in China in 2019. Notably, two of these strains had mutations in *OmpK35*, *OmpK36*, and *AcrB*, while the third strain exhibited a mutation in *AcrA*. The specific CAZ/AVI resistance-related mutations in these strains require further investigation to elucidate. The emergence of CAZ/AVI resistance in *K. pneumoniae* strains before the antibiotic’s market launch serves as a warning. It highlights the urgent need to strengthen global surveillance of antimicrobial resistance to detect and respond to potential public health risks in advance.

Bioinformatic analysis of genome sequences can be used to infer the relationship between mutations in porins and efflux pump genes and antibiotic resistance. We did not conduct a final experimental analysis and validation of the specific changes in porins and efflux pumps. Consequently, determining the expression levels of porins and efflux pumps after transcription may be challenging, which represents a limitation of our study. Our further research will focus on this aspect, where we plan to conduct transcriptomics or proteomics to verify the roles of mutations and expression of porins and efflux pumps in resistance mechanisms.

In summary, our study highlights that, alongside MBLs, *bla*_KPC_-variants are the main resistance-related mutations of CAZ/AVI resistance in *K. pneumoniae*, accompanied by varying degrees of changes in outer membrane porins and efflux pumps. The combination of multiple resistance-related mutations ultimately results in CAZ/AVI resistance in *K. pneumoniae*. The analysis of the molecular epidemiological characteristics and drug resistance mutations of CAZ/AVI-R *K. pneumoniae* worldwide provides a reference for formulating prevention and control strategies. We recommend active monitoring of CAZ/AVI-R *K. pneumoniae* strains and these related-mutations. Even in patients without prior exposure to CAZ/AVI, hospitals should routinely conduct AST for CAZ/AVI on *Enterobacteriaceae* isolated from clinical samples. Additionally, monitoring drug-resistant *Enterobacteriaceae* in hospital sewage is equally important. Simultaneously, it is essential to strengthen the prevention and control of nosocomial transmission of strains carrying specific drug-resistant mutations. Efforts should be made to enhance environmental cleaning and disinfection in high-risk departments. These measures are crucial for detecting early resistance, guiding treatment, and formulating public health interventions.

## Data Availability

The datasets presented in this study can be found in online repositories. The names of the repository/repositories and accession number(s) can be found in the article/**Supplementary Material**.

## Supplementary material

Supplementary Figure 1 Distribution characteristics of 1513 strains based on the phylogenetic tree. The red font indicates CAZ/AVI-R *K. pneumoniae* strains collected from PubMed and the PATRIC database. The blue font indicates CAZ/AVI-R *K. pneumoniae* strains collected in our group. The black font indicates CAZ/AVI-S *K. pneumoniae* with complete genomes from China from NCBI GenBank.

Supplementary Figure 2 Sankey diagram of CAZ/AVI-R *K. pneumoniae* strains from different countries and different isolation sources from 2003 to 2024. The other isolation sources of 60 strains include unknown sources, necrotic pancreatic tissue, hospital setting, and surveillance.

Supplementary Figure 3 Distribution characteristics of ISs in 48 CAZ/AVI-R *K. pneumoniae* strains. Different colors and height of columns represent IS types and IS copy number.

Supplementary Figure 4 Circular heatmap of the carrying average ARGs and virulence genes of 301 strains. The carrying of ARGs or virulence genes by each strain = the sum of ARGs or virulence genes carried by all strains/301.

Supplementary Table 1 Basic information of 48 CAZ/AVI-R *K. pneumoniae* strains collected by our research group.

Supplementary Table 2 Antimicrobial susceptibility test results of 48 CAZ/AVI-R *K. pneumoniae* strains collected by our research group.

Supplementary Table 3 Basic information of 253 CAZ/AVI-R *K. pneumoniae* strains from public databases.

Supplementary Table 4 The characteristics of plasmids carried by 301 CAZ/AVI-R *K. pneumoniae* strains.

Supplementary Table 5 The characteristics of insertion sequences (ISs) carried by 301 CAZ/AVI-R *K. pneumoniae* strains.

Supplementary Table 6 The prophages number carried by 301 CAZ/AVI-R *K. pneumoniae* strains.

Supplementary Table 7 The characteristics of ARGs on predicted prophages.

Supplementary Table 8 Virulence genes carriage of 301 CAZ/AVI-R *K. pneumoniae* strains.

Supplementary Table 9 The characteristics of ARGs in 301 CAZ/AVI-R *K. pneumoniae* strains.

Supplementary Table 10 Mutations in porins (*OmpK35* and *OmpK36*) and efflux pumps (*AcrA* and *AcrB*). A protein CIGAR consists of the following operators: (1) nM: Alignment match. Consuming n*3 nucleotides and n amino acids. (2) nI: Insertion. Consuming n amino acids. (3) nD: Delection. Consuming n*3 nucleotides. (4) nF: Frameshift deletion. Consuming n nucleotides. (5) nG: Frameshift match. Consuming n nucleotides and 1 amino acid. (6) nU: Phase-1 intron. Consuming n nucleotides and 1 amino acid. The cs tag encodes difference sequences. It consists of a series of operations: (1) “:[0-9]” represents number of identical amino acids. (2) [acgtn]+[A-Z*] represents “substitution: ref to query”. (3) “+[A-Z]” represents aa inserted to the reference. (4) “-[acgtn]” represents nt deleted from the reference.

Supplementary Table 11 The resistance-related mutations of CAZ/AVI-R *K. pneumoniae* strains from different collection sources.

Supplementary Table 12 The resistance-related mutations of 48 CAZ/AVI-R *K. pneumoniae* strains collected and isolated by our group.
