# Supplementary figures and images for: Global epidemiology and resistance-related mutations of ceftazidime-avibactam-resistant Klebsiella pneumoniae strains
Source: Front Cell Infect Microbiol. 2025 Sep 25;15:1645042. doi: 10.3389/fcimb.2025.1645042 (PMC12507769; doi:10.3389/fcimb.2025.1645042)

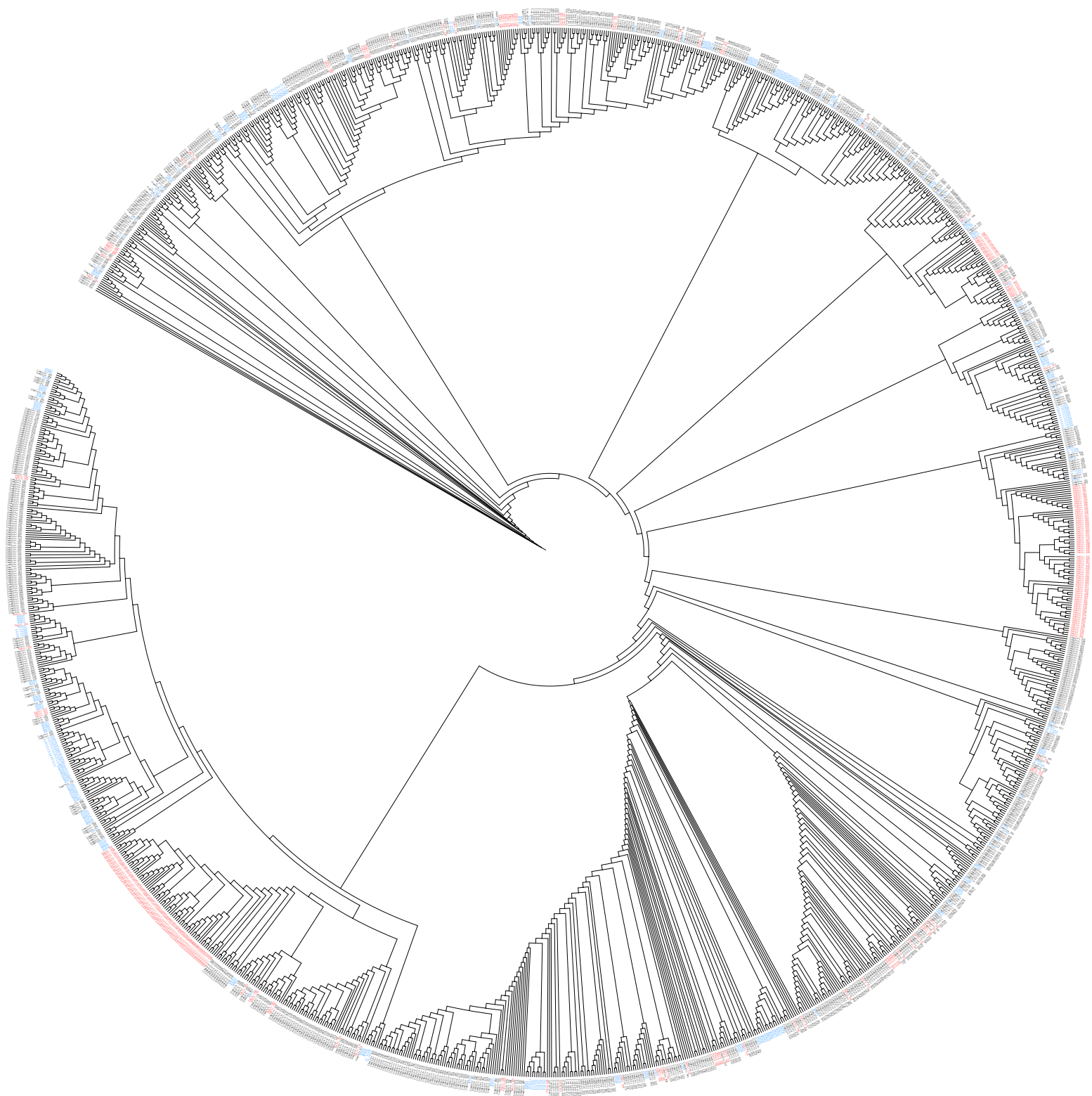

Supplement: Supplementary Figure 1 — Distribution characteristics of 1513 strains based on the phylogenetic tree. The red font indicates CAZ/AVI-R K. pneumoniae strains collected from PubMed and the PATRIC database. The blue font indicates CAZ/AVI-R K. pneumoniae strains collected in our group. The black font indicates CAZ/AVI-S K. pneumoniae with complete genomes from China from NCBI GenBank. [file Image1.pdf]

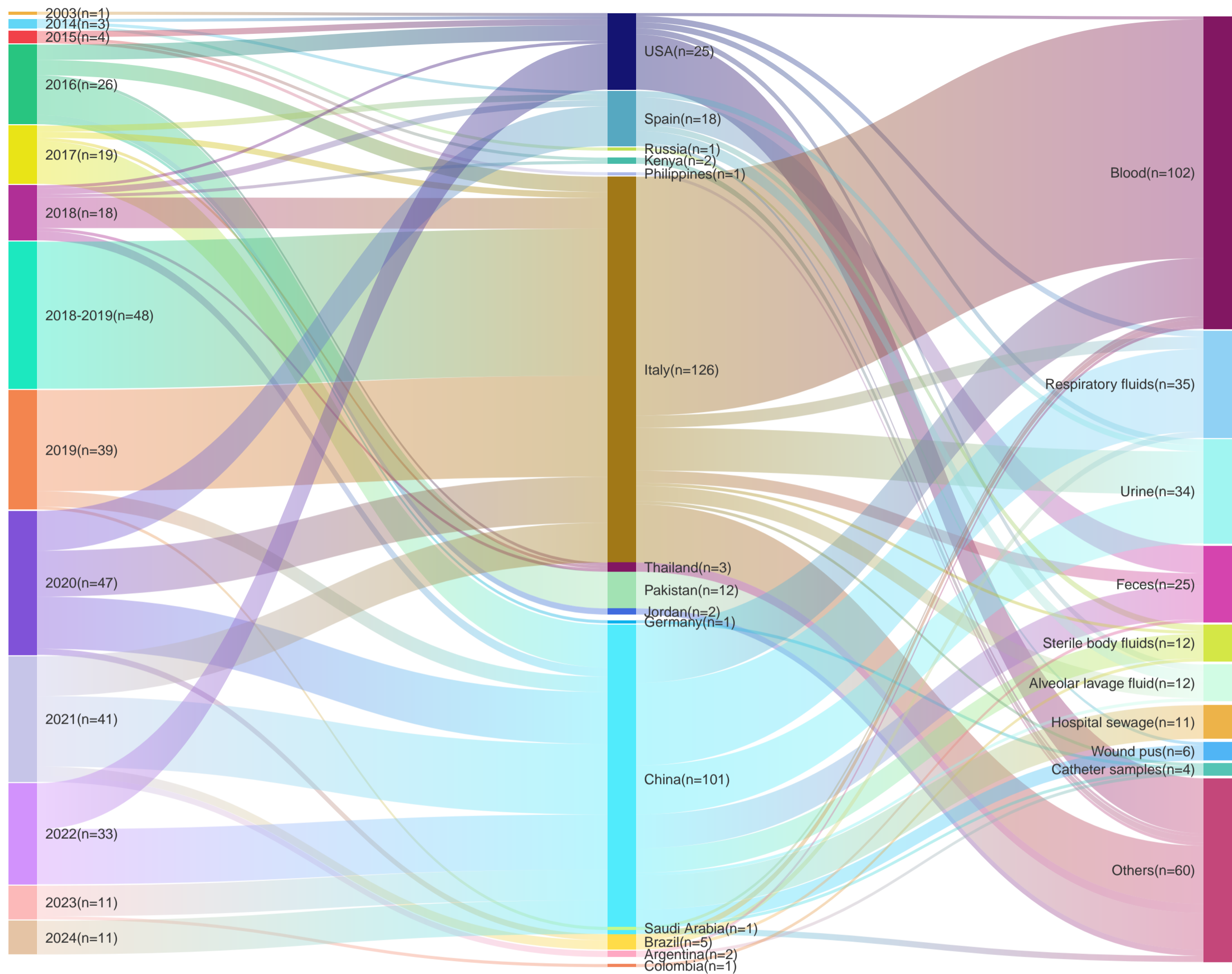

Collection Date

Geographic Location

Isolation Source

Supplement: Supplementary Figure 2 — Sankey diagram of CAZ/AVI-R K. pneumoniae strains from different countries and different isolation sources from 2003 to 2024. The other isolation sources of 60 strains include unknown sources, necrotic pancreatic tissue, hospital setting, and surveillance. [file Image2.pdf]

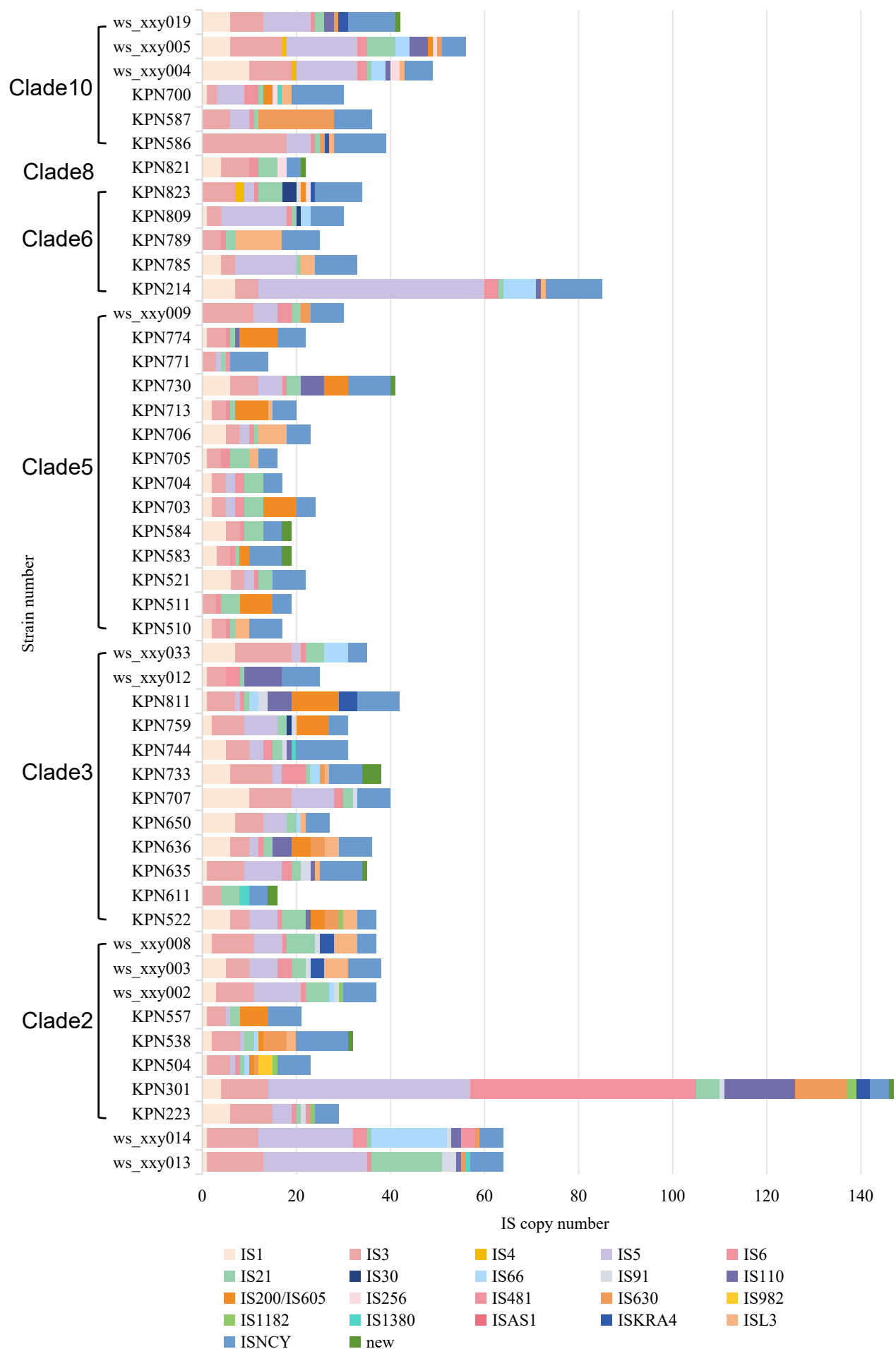

Supplement: Supplementary Figure 3 — Distribution characteristics of ISs in 48 CAZ/AVI-R K. pneumoniae strains. Different colors and height of columns represent IS types and IS copy number. [file Image3.pdf]
