# Supplementary material for: Global epidemiology and resistance-related mutations of ceftazidime-avibactam-resistant Klebsiella pneumoniae strains
Source: Front Cell Infect Microbiol. 2025 Sep 25;15:1645042. doi: 10.3389/fcimb.2025.1645042 (PMC12507769; doi:10.3389/fcimb.2025.1645042)
Supplement: Supplementary Figure 4 — Circular heatmap of the carrying average ARGs and virulence genes of 301 strains. The carrying of ARGs or virulence genes by each strain = the sum of ARGs or virulence genes carried by all strains/301. [file Image4.pdf]

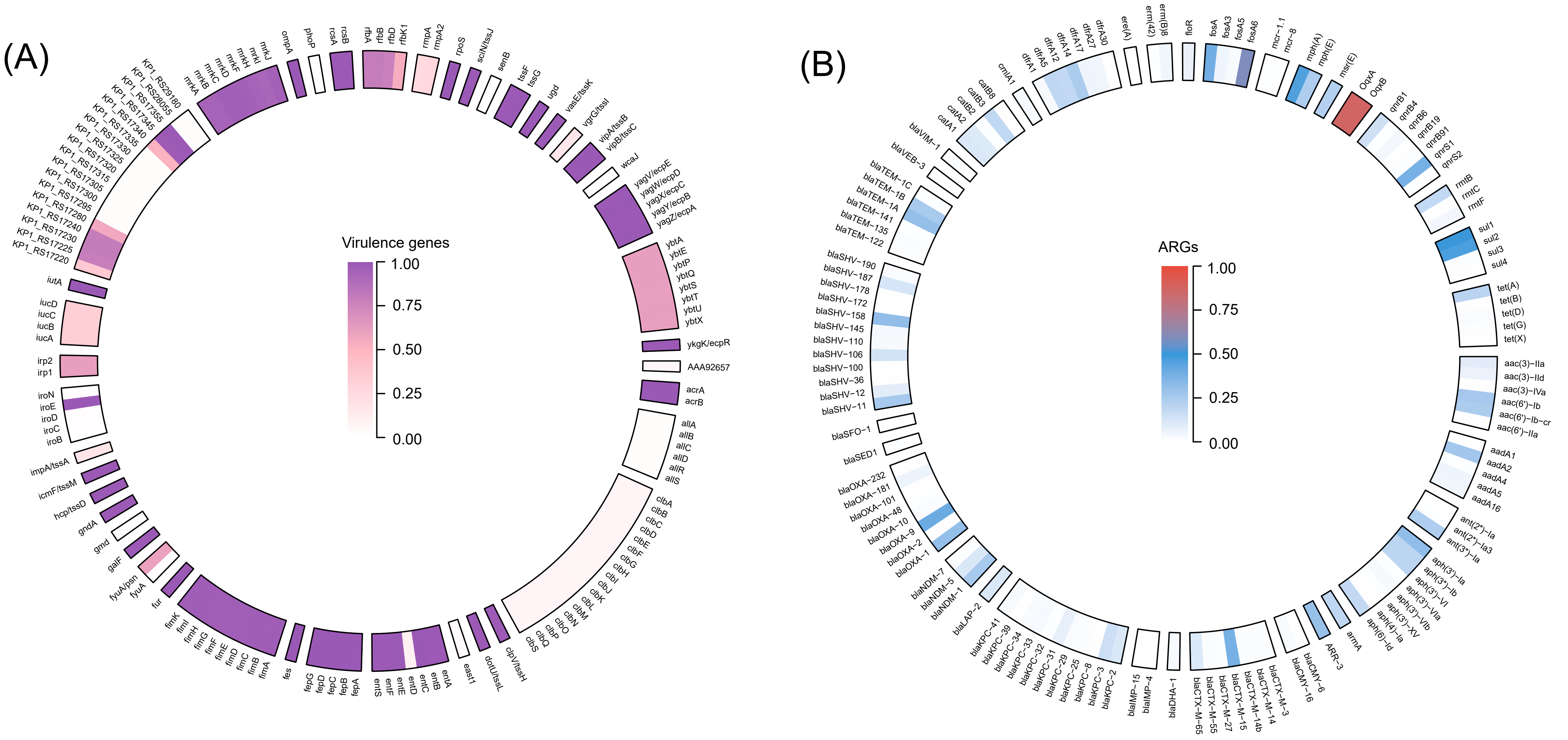
(A)
Virulence genes
1.00
0.75
0.50
0.25
0.00
KP1_RS29180
KP1_RS28055
KP1_RS17355
KP1_RS17345
KP1_RS17340
KP1_RS17335
KP1_RS17330
KP1_RS17325
KP1_RS17320
KP1_RS17315
KP1_RS17305
KP1_RS17300
KP1_RS17295
KP1_RS17280
KP1_RS17240
KP1_RS17230
KP1_RS17225
KP1_RS17220
iutA
iucD
iucC
iucB
iucA
irp2
irp1
iroN
iroE
iroD
iroC
iroB
impA/tssA
icmF/tssM
hcp/tssD
gndA
gmd
galF
fyuA/psn
fyuA
fur
fimK
fimI
fimH
fimG
fimF
fimE
fimD
fimC
fimB
fimA
fes
fepG
fepD
fepC
fepB
fepA
entS
entF
entE
entD
entC
entB
entA
east1
dotU/tssL
clpV/tssH
clbS
clbQ
clbP
clbO
clbN
clbM
clbL
clbK
clbJ
clbI
clbH
clbG
clbF
clbE
clbD
clbC
clbB
clbA
allS
allR
allD
allC
allB
allA
acrB
acrA
AAA92657
ykgK/ecpR
ybtX
ybtU
ybtT
ybtS
ybtQ
ybtP
ybtE
ybtA
yagZ/ecpA
yagY/ecpB
yagX/ecpC
yagW/ecpD
yagV/ecpE
wcaJ
vipB/tssC
vipA/tssB
vgrG/tssI
vasE/tssK
ugd
tssG
tssF
senB
sciN/tssJ
rpoS
rmpA2
rmpA
rfbK1
rfbD
rfbB
rfbA
rcsB
rcsA
phoP
ompA
mrkJ
mrkI
mrkH
mrkF
mrkD
mrkC
mrkB
mrkA
(B)
ARGs
1.00
0.75
0.50
0.25
0.00
ere(A)
erm(42)
erm(B)8
floR
fosA
fosA3
fosA5
fosA6
mcr-1.1
mcr-8
mph(A)
mph(E)
msr(E)
OqxA
OqxB
qnrB1
qnrB4
qnrB6
qnrB19
qnrB91
qnrS1
qnrS2
rmtB
rmtC
rmtF
sul1
sul2
sul3
sul4
tet(A)
tet(B)
tet(D)
tet(G)
tet(X)
aac(3)-IIa
aac(3)-IId
aac(3)-IVa
aac(6')-Ib
aac(6')-Ib-cr
aac(6')-IIa
aadA1
aadA2
aadA4
aadA5
aadA16
ant(2")-Ia
ant(2")-Ia3
ant(3")-Ia
aph(3')-Ia
aph(3")-Ib
aph(3')-VI
aph(3')-VIa
aph(3')-VIb
aph(3')-XV
aph(4)-Ia
aph(6)-Id
armA
ARR-3
blaCMY-6
blaCMY-16
blaCTX-M-3
blaCTX-M-14b
blaCTX-M-14
blaCTX-M-15
blaCTX-M-27
blaCTX-M-55
blaCTX-M-65
blaDHA-1
blaIMP-4
blaIMP-15
blaKPC-2
blaKPC-3
blaKPC-8
blaKPC-25
blaKPC-29
blaKPC-31
blaKPC-32
blaKPC-33
blaKPC-34
blaKPC-39
blaKPC-41
blaLAP-2
blaNDM-1
blaNDM-5
blaNDM-7
blaOXA-1
blaOXA-2
blaOXA-9
blaOXA-10
blaOXA-48
blaOXA-101
blaOXA-181
blaOXA-232
blaSED1
blaSFO-1
blaSHV-11
blaSHV-12
blaSHV-36
blaSHV-100
blaSHV-106
blaSHV-110
blaSHV-145
blaSHV-158
blaSHV-172
blaSHV-178
blaSHV-187
blaSHV-190
blaTEM-122
blaTEM-135
blaTEM-141
blaTEM-1A
blaTEM-1B
blaTEM-1C
blaVEB-3
blaVIM-1
catA1
catA2
catB2
catB3
catB8
cmlA1
dfrA1
dfrA5
dfrA12
dfrA14
dfrA17
dfrA27
dfrA30
